# Evaluating the Cost of Pharmaceutical Purification for a Long-Duration Space Exploration Medical Foundry

**DOI:** 10.3389/fmicb.2021.700863

**Published:** 2021-10-11

**Authors:** Matthew J. McNulty, Aaron J. Berliner, Patrick G. Negulescu, Liber McKee, Olivia Hart, Kevin Yates, Adam P. Arkin, Somen Nandi, Karen A. McDonald

**Affiliations:** ^1^Center for the Utilization of Biological Engineering in Space (CUBES), Berkeley, CA, United States; ^2^Department of Chemical Engineering, University of California, Davis, Davis, CA, United States; ^3^Department of Bioengineering, University of California, Berkeley, Berkeley, CA, United States; ^4^Global HealthShare Initiative, University of California, Davis, Davis, CA, United States

**Keywords:** techno-economic analysis, equivalent system mass, space exploration medical foundry, *in situ* resource utilization, pharmaceutical foundry, monoclonal antibody purification, human exploration mission, space systems bioengineering

## Abstract

There are medical treatment vulnerabilities in longer-duration space missions present in the current International Space Station crew health care system with risks, arising from spaceflight-accelerated pharmaceutical degradation and resupply lag times. Bioregenerative life support systems may be a way to close this risk gap by leveraging *in situ* resource utilization (ISRU) to perform pharmaceutical synthesis and purification. Recent literature has begun to consider biological ISRU using microbes and plants as the basis for pharmaceutical life support technologies. However, there has not yet been a rigorous analysis of the processing and quality systems required to implement biologically produced pharmaceuticals for human medical treatment. In this work, we use the equivalent system mass (ESM) metric to evaluate pharmaceutical purification processing strategies for longer-duration space exploration missions. Monoclonal antibodies, representing a diverse therapeutic platform capable of treating multiple space-relevant disease states, were selected as the target products for this analysis. We investigate the ESM resource costs (mass, volume, power, cooling, and crew time) of an affinity-based capture step for monoclonal antibody purification as a test case within a manned Mars mission architecture. We compare six technologies (three biotic capture methods and three abiotic capture methods), optimize scheduling to minimize ESM for each technology, and perform scenario analysis to consider a range of input stream compositions and pharmaceutical demand. We also compare the base case ESM to scenarios of alternative mission configuration, equipment models, and technology reusability. Throughout the analyses, we identify key areas for development of pharmaceutical life support technology and improvement of the ESM framework for assessment of bioregenerative life support technologies.

## Introduction

### The Need for a Pharmaceutical Foundry in Space

Surveying missions to Mars, like the InSight lander^1^ (Overview | Mission--NASA’s InSight Mars Lander) launched in 2018 and Perseverance rover^2^ in 2020, directly support the objectives of NASA’s long-term Mars Exploration Program^3^ : an effort to explore the potential for life on Mars and prepare for human exploration of Mars. The maturation of the program requires redefining the risks to human health as mission architectures transition from the current ‘‘Earth Reliant’’ paradigm used on the International Space Station (ISS) to the cislunar space ‘‘Proving Grounds’’ and finally to deep-space ‘‘Earth Independent’’ mission architectures, as defined in NASA’s report titled, ‘‘Journey to Mars: Pioneering Next Steps in Space Exploration.’’^4^

Human missions to Mars will be ‘‘Earth Independent,’’ meaning there will be very limited emergency evacuation and re-supply capabilities along with substantially delayed communications with the Earth-based mission team. The NASA Human Research Roadmap^5^ currently rates most human health risks, which include “risk of adverse health outcomes and decrements in performance due to inflight medical conditions” and “risk of ineffective or toxic medications during long-duration exploration spaceflight,” as either medium or high risk for a Mars planetary visit/habitat mission. Risk ratings are based on failure mode and effects analysis and on hazard analysis using dimensions of severity, occurrence, and detectability. A recent review highlights the current understanding of the primary hazards and health risks posed by deep space exploration as well as the six types of countermeasures: protective shielding, biological and environmental temporal monitoring, specialized workout equipment, cognition and psychological evaluations, autonomous health support, and personalized medicine (Afshinnekoo et al., 2020).

Of these countermeasures, it could be argued that medicine is the most crucial and least advanced toward mitigating space health hazards. There is very limited information on, and few direct studies of, pharmaceutical usage, stability, and therapeutic efficacy (i.e., pharmacokinetics, pharmacodynamics) in spaceflight or in a Mars surface environment (Blue et al., 2019). Furthermore, flown stores of pharmaceuticals face two additional barriers: (1) radiation-accelerated degradation (Du et al., 2011), and (2) addressing a myriad of low occurrence and high impact health hazards without the ability to fly and maintain potency of therapeutics for all of them. In these circumstances, it is often more beneficial to build robustness to these low occurrence health hazards rather than to try to predict them. It is therefore imperative that on-planet and/or in-flight pharmaceutical production be developed to bridge this risk gap. These pharmaceutical foundry technologies will supplement, not replace, the flown pharmaceutical formulary designed to treat anticipated medical threats during space missions.

Pharmaceuticals are produced either chemically or biologically. A recent review of pharmaceutical production for human life support in space compares these two methods, highlighting the need for biological production in order to address many low occurrence and high impact health hazards (e.g., sepsis, ear infection, and glaucoma) and further comparing different biological production systems (McNulty et al., 2021). One major advantage of biological production is the efficiency in transporting and synthesizing genetic information as the set of instructions, or sometimes the product itself, to meet the therapeutic needs for a variety of disease states. The emerging field of Space Systems Bioengineering (Berliner et al., 2020) encapsulates this need for biological production, of which pharmaceuticals is identified as an important subset.

### The Bottleneck of Space Foundries: Purification

Biopharmaceuticals must be purified after accumulation with the biological host organism, or cell-free transcription-translation reaction, in order to meet requirements for drug delivery and therapeutic effect (Harrison et al., 2015). The majority of commercial biopharmaceutical products are administered via intravenous and subcutaneous injection (Škalko-Basnet, 2014). Biopharmaceutical formulations for injection requires high purity (>95%) product, as impurities introduced directly into the bloodstream can trigger significant immune responses and reduce efficacy (Haile et al., 2015).

Downstream processing of biopharmaceuticals is therefore usually a resource-intensive section of overall processing, being cited as high as 80% of production costs (and contributions of input mass) for monoclonal antibody (mAb) therapeutics produced using mammalian cell cultures (Rathore et al., 2015; Budzinski et al., 2019). In addition to the processing burden for biopharmaceutical injectables, there are also often substantial storage costs involving complex supply chain and storage management with stability requirements for factors including temperature, time, humidity, light, and vibration (Sykes, 2018). There are several approaches being pursued to overcome the challenges and costs associated with downstream processing and formulation.

First are the tremendous efforts in process intensification (Strube et al., 2018). While the highly sensitive nature of biopharmaceuticals to minor process changes has introduced barriers and complexities to innovation through process intensification that have not been realized in non-healthcare biotechnological industries, there have been significant strides made in the past decade in the areas of process integration (Steinebach et al., 2017), automation (Pollard et al., 2017), and miniaturization (Adiga et al., 2018; Crowell et al., 2018).

Another route that researchers are pursuing to reduce downstream processing costs and resources is a biological solution to processing technology. In the same vein that the biopharmaceutical industry sprung out of researchers leveraging the power of biology to produce therapeutically relevant molecules that were inaccessible or excessively costly by means of chemical synthesis, researchers are now also trying to apply that same principle to purifying therapeutically relevant molecules. The simplicity of production, reagents that can be produced using self-replicating organisms, and potential recyclability of spent consumables are significant advantages of biological purification technology for space or other limited resource applications. Examples of primary biological technologies include fusion tags (Bell et al., 2013), stimuli-responsive biopolymers (Sheth et al., 2014), hydrophobic nanoparticles (Jugler et al., 2020), and plant virus nanoparticles (Werner et al., 2006; Uhde-Holzem et al., 2016).

Lastly, there are vast efforts to establish alternative drug delivery modalities (Anselmo et al., 2018). Other modalities that do not require injection and which might be more compatible to administration in limited resource environments, such as oral consumption, nasal spray, inhalation, and topical application, have long presented challenges in biopharmaceutical stability (e.g., denaturation in stomach acid) and delivery to the active site (e.g., passing the gut-blood barrier) that minimize product efficacy and necessitate costly advanced formulations and chemistries (Mitragotri et al., 2014).

A particularly promising drug delivery technique to circumvent downstream processing burdens is to sequester the active pharmaceutical ingredient in the host cells of the upstream production system as a protective encapsulation in order to facilitate bioavailability through oral delivery (Kwon and Daniell, 2015). It represents an opportunity to greatly lower the cost of *in situ* production of human medicine for a space mission. This technique presumes that the host system is safe for human consumption, and so naturally lends itself to utility in systems such as yeast and plant production hosts. Oral delivery via host cell encapsulation has been recently established as commercial drug delivery modality with the US Food and Drug Administration approval of Palforzia as an oral peanut-protein immunotherapy (Vickery et al., 2018). However, this solution is not necessarily amenable to the diversity of pharmaceutical countermeasures that may be required, especially for unanticipated needs in which the product may not have been evaluated for oral bioavailability.

### Space Economics

In 2011, the space shuttle program was retired due to increasing costs, demonstrating that reduction of economic cost is critical for sustaining any campaign of human exploration (Wall, 2011). Although recent efforts in reducing the launch cost to low earth orbit by commercial space companies have aided in the redefinition of the space economy (Whealan George, 2019), the barrier to longer term missions, such as a journey to Mars, is still limited by the extreme financial cost in transporting resources. Additionally, it has been shown that as the mission duration and complexity increases–as expected for a human mission to Mars–the quantity of supplies required to maintain crew health also increases (Anderson et al., 2018). In the case of meeting the demand for medication, biopharmaceutical synthesis has been proposed as an alternative to packaging a growing number of different medications (Menezes et al., 2015; McNulty et al., 2021). Assuming that both technologies can meet mission demand, selection of the production-based biotechnology platform will be dependent on its cost impact. It is therefore critical that the cost model of biopharmaceutical synthesis accounts for and minimizes the cost of any and all subprocesses, including those for purification.

The current terrestrial biopharmaceutical synthesis cost model does not align with the needs for space exploration environments. For example, the literature highlights the high cost of Protein A affinity chromatography resin ($8,000–$15,000/L) and the need to reduce the price (Ramos-de-la-Peña et al., 2019). However, the purchase cost of chromatography resin is not nearly as critical in space environment applications where the major costs are more closely tied to the physical properties of the object (mass, volume, refrigeration requirements, etc.), as a result of fuel and payload limitations and the crew time required for operation (Jones, 2001). The distinct cost models of space and terrestrial biopharmaceutical production may increase the burden of identifying space-relevant processing technologies and may also limit direct transferability of terrestrial technologies without attention given to these areas.

On the other hand, changing incentives structures relating to sustainability and the advent of new platform technologies are rapidly increasing alignment and the potential for technology crossover. For example, companies like On Demand Pharmaceuticals^6^, EQRx^7^, and the kenUP Foundation^8^, initiatives leading to industry adoption of environmental footprint metrics such as E-factor (Sheldon, 2007) and process mass intensity (PMI) (Budzinski et al., 2019), and diffusion from the adjacencies of green and white biotechnology (Tylecote, 2019) all promote development of accessible and sustainable technologies. As these trends pertain to space-relevant processes, these examples can also be viewed as driving more closed loop systems composed of simpler components.

#### Reference Mission Architecture

The evaluation of biopharmaceutical system cost for space applications requires the establishment of a reference mission architecture (RMA) as a means for describing the envelope of the mission scenario and distilling initial technology specifications which relate to the proposed subsystem in question (Drake and Watts, 2014). This RMA can be used to orient and define the specific mission elements that meet the mission requirements and factor into the calculations of cost for deploying biopharmaceutical technologies. Ultimately, the RMA provides the means to determine and compare cost given specification of mission scenarios that utilize the technology in question. We envision developing and integrating biotechnological capabilities back-ended by purification and quality systems into standard methods composed of a series of unit procedures that maintain astronaut health via the Environmental Control and Life Support Systems (ECLSS) (Hendrickx et al., 2006). In this study, we begin to build toward this vision by proposing a high-level RMA that specifies a biopharmaceutical demand partially fulfilled through biomanufacturing over the course of a defined production window.

#### Equivalent System Mass

In planning for future human exploration missions, technology choices and life-support systems specifications are often evaluated through the metric of the equivalent system mass (ESM) (Levri et al., 2003). Driven by the economic factor of cost in dollars required to transport mass into orbit, the ESM framework accounts for non-mass factors such as power, volume, and crew-time by relating them to mass through predetermined equivalency factors. ESM has been used to evaluate the mass of all of the resources of a larger system including water, shielding materials, agriculture and recycle loop closure. Currently, ESM remains the standard metric for evaluating advanced life support technology platforms (Hogan et al., 2000; Zabel, 2020). In the Space Systems Bioengineering context of realizing a biomanufactory on the surface of Mars (Berliner et al., 2020), recent advances in extending this metric have been proposed in the form of extended ESM which attempts to address complexities stemming from multiple transit and operations stages, as would be required to support a crewed mission to Mars (Berliner et al., 2021). It also accounts for uncertainties inherent in mission planning such as technology failures and their downstream effects as propagated through a mission such as refrigeration failures in systems housing medicine that requires specific cooling. Such advances in the ESM framework aid in the assessment of biopharmaceutical technologies as elements in the context of proposed ECLSS given the inherent stochastic nature of human health, especially in a space environment (Bizzarri et al., 2017). Here, we calculate ESM at multiple mission segments across which biopharmaceutical purification is deployed.

## Materials and Methods

### Unit Procedure Selection

#### Protein A-Based Affinity Capture Step

The medical significance of mAb therapies and the highly developed and specialized purification technology provide a fertile ground for techno-economic feasibility analysis of an *in situ* resource utilization (ISRU)-based pharmaceutical foundry for space. The first reason is that there are mAb therapies commercially approved or in development for multiple important disease states of spaceflight including osteoporosis (Faienza et al., 2018), migraines/headaches (Schuster and Rapoport, 2016), seizure (Zhao et al., 2017), pneumonia (Hua et al., 2014), ocular herpes (Krawczyk et al., 2015), otitis media (Iino et al., 2019), various oncological indications (Zahavi and Weiner, 2020), and fungal infections (Ulrich and Ebel, 2020). A second reason is that degradation products of mAb therapies are known to result in, not just reduced efficacy, but also deleterious effects (e.g., harmful immune reactions in patients) that further compound concerns of pharmaceutical stability over a long-duration mission (Laptoš and Omersel, 2018). Thirdly is that a common manufacturing system can be used to produce treatments for a variety of indications which is highly advantageous in mass and volume savings for spaceflight. And fourthly, the economic incentive of research into mAb purification technology has resulted in a plethora of technologies, enabling this analysis to include head-to-head comparisons between multiple mAb capture steps of different origins (e.g., biotic, abiotic) and different processing mechanisms (e.g., bind-and-elute mode liquid chromatography, precipitation). It is in comparing the differences between these technologies that we can uncover general insights into the desired components of a pharmaceutical foundry for space.

Monoclonal antibody therapy is a platform technology that supports human health across a diversity of medical indications with a generally maintained molecular structure, in large part due to the coupling of high target selectivity in the two small and highly variable complementarity-determining regions located in the antigen-binding fragments (Goding, 1996) and control of the biological action on that target (i.e., effector function) through the generally conserved fragment crystallizable (Fc) region (Kang and Jung, 2019). This otherwise high structural fidelity conserved across mAb therapy products (which are primarily of the immunoglobulin G class) spans a wide variety of therapeutic indications and creates an opportunity for generic mAb production process flows, which include technologies devised specifically for mAb production (Sommerfeld and Strube, 2005). This specialized manufacturing, which is most notable in the use of the affinity capture step targeting the Fc region of an antibody with the use of the protein-based ligands derived from the *Staphylococcus aureus* Protein A molecule, can be tuned for highly efficient purification of mAb and antibody-derived (e.g., Fc-fusion protein) class molecules (Ramos-de-la-Peña et al., 2019). Therefore, we have decided to investigate the Protein A-based affinity capture step in isolation as a starting point for understanding the costs of a potential pharmaceutical foundry in space.

It is worth noting that other similar protein ligands, such as Protein G and Protein L, are also widely used for their ability to capture different types of immunoglobulin classes and subclasses more efficiently (Choe et al., 2016).

#### Abiotic and Biotic Protein A-Based Unit Procedures

**FIGURE 1:**
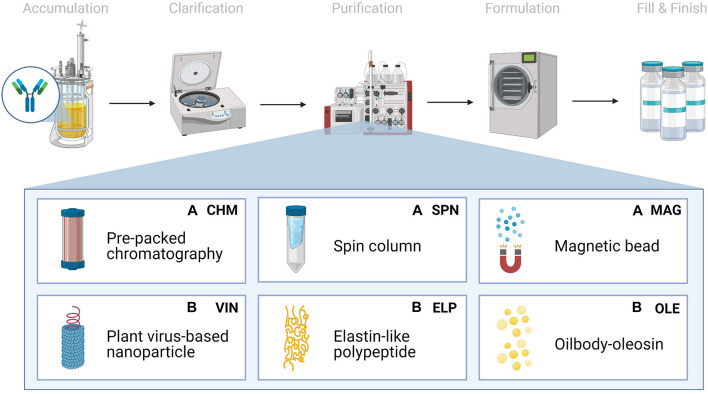
Monoclonal antibody production consists generically of product accumulation, clarification, initial purification, formulation, and fill & finish. Here we investigate six technologies for the capture step within the first purification step in a space mission context using extended equivalent system mass. The manufacturing origin of the capture reagent is denoted as either **(A)** abiotic or **(B)** biotic.

We chose to analyze six Protein A-based capture step procedures: three commercially available abiotic technologies [pre-packed chromatography (CHM), spin column (SPN), and magnetic bead (MAG)] and three development-stage biotic technologies [plant virus-based nanoparticle (VIN), elastin-like polypeptide (ELP), and oilbody-oleosin (OLE)] (Figure 1). Commercial technology procedures are based on product handbooks while the procedures of developing technologies, which we would classify as Technology Readiness Level 2 per NASA’s guidelines, are based on reports in literature. This set of procedures was selected to survey a wide range of operational modalities, technological chassis, and perceived advantages and disadvantages (Table 1).

**TABLE 1:** List of Protein A-based monoclonal antibody capture step unit procedures included for analysis.

Unit procedure ID	Method	Technology used	References
Pre-packed chromatography^(A)^	Liquid chromatography	Pre-packed HiTrap MabSelect SuRe column of novel alkali-tolerant recombinant Protein A-based ligand coupled with an agarose matrix	Vendor handbooks (Cytiva, 2006, 2020, 2021)
Spin column^(A)^	Centrifuge-assisted liquid chromatography	Pre-packed Protein A HP SpinTrap spin column containing Protein A Sepharose High Performance	
Magnetic bead^(A)^	Magnetic separation	Protein A Mag Sepharose superparamagnetic beads coupled with native Protein A ligands	
Plant virus-based nanoparticle^(B)^	Sedimentation complex	Plant virion, *Turnip vein clearing virus*, presenting a C-terminal coat protein fusion display of Protein A (domains D and E)	Werner et al., 2006
Elastin-like polypeptide^(B)^	Inverse transition cycle	Elastin-like polypeptides [78 pentapeptide (VPGVG) repeats] fused with Z domain, an engineered B domain of Protein A	Sheth et al., 2014
Oilbody-oleosin^(B)^	Liquid-liquid partition	*Arabidopsis* oleosin fused at the N-terminal with an engineered Protein A(5)	McLean et al., 2012

*^*A*^Abiotic technology. ^*B*^Biotic technology.*

All six of the unit procedures are operated in bind-and-elute mode, in which a clarified mAb-containing liquid stream is fed into a capture step containing Protein A-based ligand, which selectively binds the mAb and separates the mAb from the bulk feed stream. The mAb is eluted from the Protein A-based ligand and recovered using a low pH buffer to dissociate the mAb from the ligand. Finally, the low pH environment of the recovered mAb is pH neutralized for future processing or storage. The analysis does not consider differences in mAb processing upstream or downstream of the affinity capture step that may arise from differences in the unit procedure operations.

Pre-packed chromatography is a chromatography system consisting of a liquid sample mobile phase which is pumped through a pre-packed bed of Protein A-fused resin beads housed in a column. SPN is a similar system, in which a Protein A-fused resin bead bed has been pre-packed into a plastic tube housing and the mobile phase flow is controlled via centrifugation of the plastic tube. MAG is a slurry-based magnetic separation system that uses superparamagnetic particles coated with Protein A-fused resin mixed as a slurry with the feed mAb stream for capture and elution of the mAb by magnet. VIN is a sedimentation-based system that uses plant virion-based chassis fused with Protein A-based ligands in suspension for capture of the mAb and centrifugation, assisted by the sedimentation velocity contribution of the chassis, to isolate and elute the mAb. ELP is a precipitation-based system that uses stimuli-responsive biopolymers fused with Protein A-based ligands in suspension for capture of the mAb and external stimuli (e.g., temperature, salt) to precipitate the bound complex and elute the mAb. OLE is a liquid-liquid partitioning system that uses oil phase segregating oleosin proteins fused with Protein A-based ligands to capture mAb in the oil phase and then elute the mAb into a clean aqueous phase.

### Techno-Economic Evaluation

Techno-economic evaluations are performed using the recently proposed equations for ESM that include calculation of costs at each mission segment (Berliner et al., 2021). ESM for the mission ESM_0_ is defined as


$${\text{ESM}}_{0}=\sum_{k}^{ℳ}L_{e\,q,k}\,\sum_{i}^{A_{k}}\left[\left(M_{k_{i}}\cdot M_{e\,q,k}\right)+\left(V_{k_{i}}\cdot V_{e\,q,k}\right)+\left(P_{k_{i}}\cdot P_{e\,q,k}\right)+\left(C_{k_{i}}\cdot C_{e\,q,k}\right)+\left(T_{i}\cdot D_{k}\cdot T_{e\,q,k}\right)\right]={\text{ESM}}_{0,p\,d}+{\text{ESM}}_{0,t\,r\,1}+{\text{ESM}}_{0,s\,f}+{\text{ESM}}_{0,t\,r\,2}$$

where *M_i_*, *V_i_*,*P_i_*,*C_i_*, *T_i_* are the initial mass [kg], volume [m^3^], power requirement [kW], cooling requirement [kg/kW], and crew-time requirement [CM-h/h], *M*_*eq*_, *V*_*eq*_,*P_eq_*,*C_eq_*, *T*_*eq*_ are the equivalency factors for mass [kg/kg] (which is set to 1 in this study), volume [kg/m^3^], power [kg/kW], cooling [kg/kW], and crew time [kg/CM-h], respectively, *L*_*eq*_ is the location equivalency factor [kg/kg] that accounts for costs associated with mass transport occurring at a particular mission segment (e.g., orbital maneuvers required for the return transit), and *D* is the duration of the mission segment [day] over a set of subsystems *i* ∈ *A* and set of mission segments *k* ∈ℳ. The mission ESM in this study is specifically defined as the sum of subtotal ESM for each mission segment within the scope of the RMA defined in this study (pre-deployment ESM_0,*pd*_, crewed transit to Mars ESM_0,*tr*1_, Mars surface operations ESM_0,*sf*_, and return crewed transit to Earth ESM_0,*tr*2_).

**FIGURE 2:**
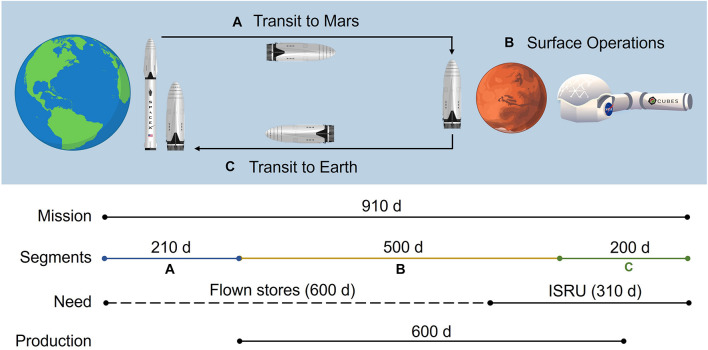
An illustration of the reference mission architecture in which **(A)** a crewed ship is launched from the surface of Earth and lands on Mars and **(B)** assembles a pre-deployed habitat on the Martian surface to perform operations before **(C)** a return transit to Earth on the same ship. Pharmaceutical needs are supported by flown stores until partway through surface operations, at which point needs are met by pharmaceuticals produced using *in situ* resource utilization. Production is initiated prior to the need window to ensure adequate stocks are generated by the time it is needed. Rocket artwork adapted from Musk (2017). Habitat artwork by Davian Ho.

Key mission and pharmaceutical assumptions are summarized in Table 2. The mission timeline depicted in Figure 2 provides insight into the proposed RMA and downstream crew needs and mAb production horizon. Here we assume a total mission duration of 910 days. First, a crew of 6 will travel from Earth to low Earth orbit, then board an interplanetary craft for a 210-day journey to Martian orbit, where the crew will descend to the surface in a separate craft, allowing the large transit vehicle to remain in orbit. Once on Mars, the crew will perform surface operations for 600 days. Following surface operations, the crew will leave Mars in a fueled ascent craft, board the interplanetary vehicle, and return to Earth orbit in 200 days. The mission timeline, crew size, and ESM equivalency factors are consistent with the recent RMA presented for inclusion of biomanufacturing elements (Berliner et al., 2021).

**TABLE 2:** Key mission and pharmaceutical reference mission architecture details and assumptions.

Mission scope	
Pre-deployment	N/A
Transit to Mars	210 days
Surface operations	500 days
Return transit	200 days
Total mission duration	910 days
Crew size	6 crew members

**Pharmaceutical scope**	

Mission demand, mAb	30,000 mg
Biomanufacturing, mAb	10,000 mg
Capture step recovery	98%
Production window	600 days
Feed mAb concentration	1 mg/mL
Molecular weight, mAb	150 kDa

*mAb, monoclonal antibody.*

The mission demand for mAb therapies is assumed to be 30,000 mg over the entirety of the mission (supporting logic detailed in Supplementary Table 1). Pharmaceutical stores and production resources are assumed to be flown with the crew transit (no pre-deployment in order to maximize shelf-life). We assume that the production resources are stable throughout the mission duration. We conservatively assume (in the face of insufficient spaceflight stability data for biologics for a more refined estimate) that the first 600 days of pharmaceutical demand will be met through flown stores (20,000 mg), at which point pharmaceutical ISRU manufacturing is needed (10,000 mg) to alleviate the impact of accelerated pharmaceutical degradation and provide supplementary medication. The pharmaceutical production window opens prior to the ISRU demand timeframe and persists through a portion of the return transit (up to mission day 810) to reflect the expected life support advantage of maintaining capabilities to counter unanticipated needs or threats. We assume that the Protein A-based unit procedures consistently yield 98% recovery of mAb from the input stream.

### Unit Procedure Simulation

Deterministic models for each unit procedure were developed in Microsoft Excel (Supplementary Data Sheet 2) using reference protocols cited in Table 1 as a series of executable operations, each containing a set of inputs defined by cost categories (labor, equipment, raw materials, and consumables) that are correspondingly populated with characteristic ESM constituent (mass, volume, power, cooling, and labor time) values (model composition illustrated in Supplementary Figure 1). Unit procedures have been defined as the smallest single execution (i.e., unit) of the secondary purification capture step procedure according to the reference protocol. We define the unit capacity by volume according to the equipment and consumables used (e.g., 2 mL maximum working volume in a 2 mL tube) and by mAb quantity according to the binding capacity for the given method (e.g., 1 mg mAb/mL resin) (Supplementary Table 2). Unit procedures with no explicit working volume constraints (i.e., the liquid solution volume for biotic technologies) have been defined with a maximum unit volume of 2 mL. ESM-relevant characteristics of individual inputs (e.g., equilibration buffer, 2 mL tube) are defined based on publicly available values, direct measurements taken, and assumptions (which are explicitly identified in the Supplementary Data Sheet 2).

There are several model features that we have considered and decided not to include within the scope of analysis. Packing and containers for the inputs are not included for three reasons: (1) the contributions of the container are considered negligible as compared to the input itself (e.g., container holding 1 L buffer as compared to the 1 L of liquid buffer); (2) materials flown to space are often re-packaged with special considerations (Wotring, 2018); and (3) the selection of optimal container size is non-trivial and may risk obscuring more relevant ESM findings if not chosen carefully. We do not consider buffer preparation and assume the use of flown ready-to-use buffers and solutions. Furthermore, refrigeration costs of the input materials and costs that may be associated with establishing and maintaining a sterile operating environment (e.g., biosafety cabinet, 70% ethanol in spray bottles) are expected to be comparable between unit procedures and not considered. Impacts of microgravity on unit procedure execution are not considered for the return transit production. Refrigeration costs associated with low temperature equipment operation (e.g., centrifugation at 4°C) are included in the equipment power costs.

Inputs common across unit procedures are standardized (Supplementary Table 3). One operational standardization is the inclusion of pH neutralization of the product stream following the low pH elution mechanism, which was explicitly stated in some procedures while not in others. Input quantities are scaled from a single unit to determine the number of units required to meet the RMA specifications. The ESM constituent inputs (mass, volume, power, cooling, and labor time) are converted into equivalent mass values using RMA equivalency factors (Supplementary Table 4).

## Results and Discussion

### Standardization of Manufacturing Efficiency

**FIGURE 3:**
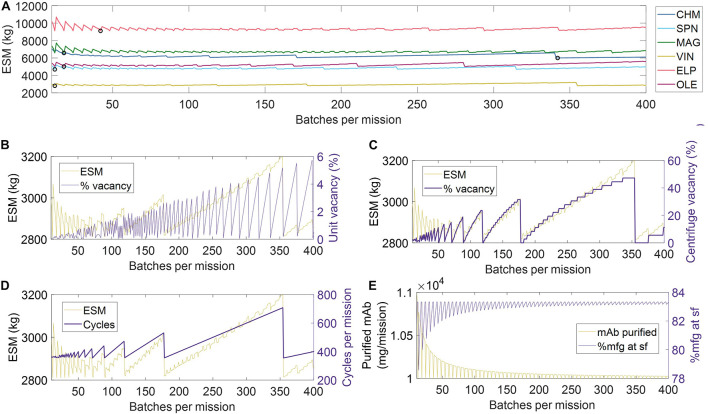
**(A)** Scheduling optimization for the establishment of base case scenarios for each unit procedure. The value for number of batches corresponding to the minimum equivalent system mass for each unit procedure, as indicated by black circle (∘) markers. Key operational parameters impacted by mission scheduling (shown using the VIN procedure) include **(B)** unit underutilization or vacancy, **(C)** equipment underutilization or vacancy, in this case represented by the centrifuge as the bottleneck, **(D)** the number of use cycles, and **(E)** the total quantity of monoclonal antibody (mAb) per mission and per surface operation (sf). CHM, pre-packed chromatography; SPN, spin column; MAG, magnetic bead; VIN, plant virus-based nanoparticle; ELP, elastin-like polypeptide; OLE, oilbody-oleosin.

Given the limited granularity of the presented RMA, which was scoped as such to reflect the lack of literature presenting an overarching and validated Concept of Operations for a Transit to Mars (Antonsen et al., 2017), we do not define strict manufacturing scheduling criteria for pharmaceutical production. Construction of a detailed pharmaceutical production RMA is hindered by uncertainty in the number and identity of mAb therapy products that would be included within mission scope, the decay rate of mAb therapy stores in the mission environments, and a reasonable basis for building robustness to unanticipated disease states. Rather, we choose to establish an objective comparison between unit procedures by normalizing for scheduling-associated manufacturing efficiencies. We accomplish this by first identifying the number of batches per mission (and thus batch size) needed to meet the mAb demand (base case of 10,204 mg mAb feed assuming 98% recovery) that minimizes the ESM output for a given unit procedure, and then running the simulation of pharmaceutical production at that number of mission batches, as shown in Figure 3A and tabulated in Supplementary Table 5.

In Figures 3B–E, we visualize a deconstruction of ESM output, using the VIN unit procedure as an example, by key performance metrics that vary with a scheduling dependence in order to illustrate the significance of batch optimization in unit procedure comparison. The processing of a given batch volume and mAb quantity is allocated into a number of units, as determined by the volume and mAb quantity constraints of a given unit procedure, and a number of use cycles per batch, as determined by the capacity of the equipment specified in the given unit procedure. We show how the variation in ESM output over the number of mission batches maps to extent of unit vacancy or underutilization (Figure 3B), extent of operational equipment (e.g., centrifuge) vacancy or underutilization (Figure 3C), and number of required use cycles (Figure 3D). We also show an oscillatory behavior in the scheduling (i.e., total mAb purified per mission, % purified at surface operations) that quickly dampens as number of mission batches increases (Figure 3E). This behavior is a result of the assumption that the mAb feed stream is coming from a discrete upstream production batch (e.g., batch-mode bioreactor) that does not output partial batch quantities, as opposed to a continuous upstream production for which there are no defined batches. Accordingly, partial batch needs are met by the processing of a full batch.

### Base Case Scenario

**FIGURE 4:**
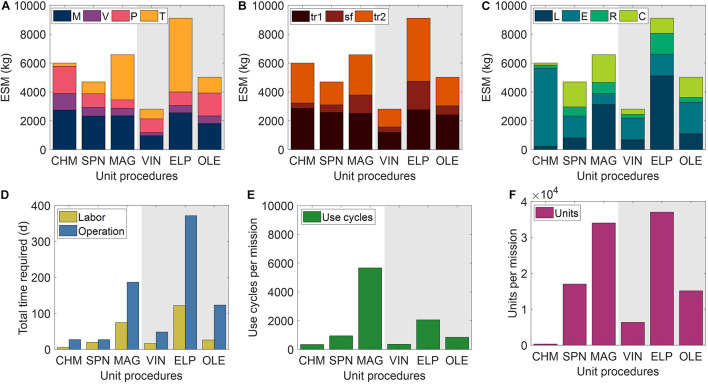
Base case equivalent system mass results broken down by **(A)** mass (M), volume (V), power (P), and labor time (T) constituents, **(B)** transit to Mars (tr_1_), surface operations (sf), and return transit (tr_2_) mission segments, and **(C)** labor (L), equipment (E), raw materials (R), and consumables (C) cost category for the six tested Protein A-based monoclonal antibody affinity capture step unit procedures segregated by abiotic (white background) and biotic (gray background) technologies. Also shown are the **(D)** labor and operation times, **(E)** number of use cycles, and **(F)** number of units required for each unit procedure to meet the reference mission demand. CHM, pre-packed chromatography; SPN, spin column; MAG, magnetic bead; VIN, plant virus-based nanoparticle; ELP, elastin-like polypeptide; OLE, oilbody-oleosin.

The ESM and output metrics of the base case scenario (10,000 mg mAb demand, 1 mg mAb/mL feed concentration, 98% recovery) for each of the six unit procedures are shown in Figures 4A–F. From this viewpoint of an ESM output for an isolated unit procedure outside the context of a full purification scheme, the ESM ranked from lowest to highest are VIN < SPN < OLE < CHM < MAG < OLE. However, we reason that it is more important to understand the model inputs that influence the ESM output rankings than to use the rankings in this isolated subsystem analysis to make technology selection choices, which requires the context of a full pharmaceutical foundry and of linkages to other mission elements.

We observe that mass costs are generally the primary contributor to ESM output, except for the MAG and ELP procedures in which labor time costs are larger. The mass costs are not closely associated to any given cost category across unit procedures, but rather the breakdown of mass costs varies widely by unit procedure.

Power costs (kW) are disproportionately high given that the static nature of ESM assumes constant usage, and thus energy (kW⋅h) in this context (i.e., the power supply to the equipment is not turned off in this analysis). These costs represent an upper bound assuming that the power supply system capacity is sized to support a maximal power consumption in which all power-drawing elements are simultaneously in operation. Time of power usage as a fraction of duration are as follows: CHM (99%) > MAG (78%) > ELP (48%) > SPN (45%) > OLE (42%) > VIN (30%). The lower use fraction unit procedures are therefore paying a relatively higher cost per unit power demand in this current method. The electrical needs of the equipment used by the unit procedures are within NASA-proposed Mars mission RMA bounds, with energy use across all unit procedures would peak at ∼1% of a proposed Mars transfer vehicle electric capacity (50 kWe) or ∼5% of the habitat capacity (12 kWe) of a reference stationary surface nuclear fission power reactor (Drake et al., 2010).

The mission segment breakdown of ESM illustrates the relatively high costs of pharmaceutical manufacturing capabilities for transit, even for the transit to Mars (tr_1_) in which there is no actual production taking place. There is a strong economic incentive to limit the amount of supplies flown on tr_1_. Alternatives such as the pre-deployment of reagents and consumables and limiting of production to surface operations on Mars (which has lower RMA equivalency factors for mass and volume than transit operations) must be balanced against the risk to human health posed by removing pharmaceutical production capabilities from a mission segment and potentially exposing the supplies to longer storage times that could challenge shelf lives.

Labor and operation times are important parameters in the broader mission and pharmaceutical foundry context. These unit procedures represent a single step of pharmaceutical production, which if realized in a space mission context, would, in turn, need to be a small portion of a crew member’s time allocation. Assuming 40-h work weeks for crew members, the labor time spans a range of ∼1% (CHM) to ∼14% (ELP) of the available crew time over the 600-day production window. It is not feasible to operationalize with such high labor and operation times at this scale of production, particularly as they stand for MAG and ELP. While strategies such as batch staggering and concurrency can be used to reduce durations, advanced automation will almost certainly need to be built into the core of a pharmaceutical foundry.

A prevailing trend throughout the unit procedures is that the number of unit executions and use cycles required by a given unit procedure are positive correlated with the ESM output value, except for the equipment cost-dominant and higher unit capacity CHM procedure. The equipment modeled in the analysis for CHM and the other unit procedures are almost certainly not space-ready and could be further designed to reduce mass and volume and increase automation to reduce crew labor time. The increased equipment costs in the CHM procedure are primarily due to automation and monitoring hardware for running liquid chromatography, which is reflected in the minimal labor costs of the CHM procedure. Miniaturization efforts, such as those focusing on microfluidic systems (Millet et al., 2015; Rodríguez-Ruiz et al., 2018; Murphy et al., 2019), are emerging as a potential path toward mitigating the high equipment costs associated with highly automated and tightly controlled manufacturing, which are crucial for freeing up valuable crew time.

The number of unit executions is determined by the binding capacity of the technology and the nominal unit size. This indicates that the unit capacity for purification is an important consideration and influential factor. Unit sizing is an important consideration that is valuable to assess more holistically within the broader pharmaceutical production and mission context.

The number of use cycles is determined by the number of unit executions required and by the maximal unit capacity of the equipment items (e.g., if you presume that an 18-slot centrifuge is the equipment bottleneck then the effective number of batches is the number of units required divided by 18). Therefore, it can be understood that the equipment unit capacity is a critical parameter in tuning the number of use cycles and, by extension, the labor costs. For processes with lower labor costs, due to the intrinsic nature of the procedure or through automation of labor, equipment unit capacity will still influence the total duration and production throughout. The MAG and ELP procedures yield both high labor and duration times and are thus particularly sensitive to the equipment capacity.

#### Contextualizing Equivalent System Mass With Supporting Evaluations

Having acknowledged shortcomings of ESM as a decision-making tool for comparison of alternative approaches in isolated subsystems, we propose that supplementary evaluations can assist in contextualization. A primary gap of an isolated subsystem ESM analysis is a lack of information on the holistic usefulness or cost of a given employed resource, which could include its synergy with other mission subsystems and its extent of recyclability, or waste loop closure, within the mission context. For example, the isolated subsystem analysis does not capture information on the broad applicability that a centrifuge might have for use in other scientific endeavors, nor do the ESM outputs reflect the >93% recyclability of water achieved by the recycler on the ISS (Steven Siceloff, 2008) that may be generalizable to future missions.

**FIGURE 5:**
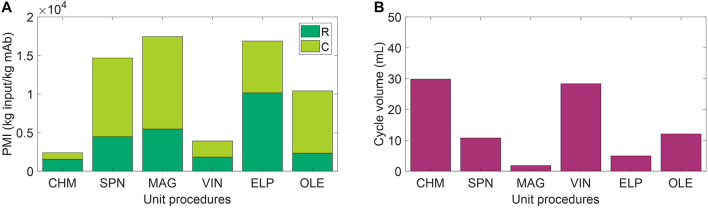
**(A)** Process mass intensity (PMI) evaluation of the unit procedures broken down by raw materials (R) and consumables (C) contributions. **(B)** Cycle volume for each unit procedure. CHM, pre-packed chromatography; SPN, spin column; MAG, magnetic bead; VIN, plant virus-based nanoparticle; ELP, elastin-like polypeptide; OLE, oilbody-oleosin.

The use of environmental footprint metrics, such as PMI, may be one valuable step toward capturing missed information on recyclability. PMI is a simple metric of material efficiency defined as the mass of raw materials and consumables required to produce 1 kg of active pharmaceutical ingredient. The study by Budzinski et al. (2019) introducing PMI for biopharmaceuticals presents data from 6 firms using small-scale (2,000–5,000 L reactor) and large-scale (12,000–20,000 L reactor) mAb manufacturing operations, finding an average 7,700 kg of input is required to produce 1 kg of mAb. Figure 5A presents PMI evaluation for the six capture steps included in analysis, which result in PMI outputs as low as 2,390 kg of input (CHM) and as high as 17,450 kg of input (MAG) per 1 kg of mAb. A comparison of these outputs to those of Budzinski et al. (2019) indicates that we may be observing roughly similar values after accounting for the high cost of initial purification in the study, representing ∼60% of the total PMI reported, the elevated feed mAb concentration (i.e., cell culture titer) of 1–5.5 g mAb/L, and adjustments for economies of scale when operating at such low cycle volumes (Figure 5B). Consumable costs appear to be the most sensitive to scale, which represents ∼1% total PMI on average in the values reported by Budzinski et al. (2019) and ranges from 35% (CHM) to 77% (OLE) here. Budzinski et al. (2019) also go one step further to distinguish water as a separate category from raw materials and report that >90% of the mass is due to water use. Here we assume pre-made buffers and do not directly add water in this study, so we refrain from a similar calculation, but it is worth noting that the extent of water use may also serve as a reasonable starting surrogate for extent of achievable recyclability in a space mission context.

### Scenario Analysis

**FIGURE 6:**
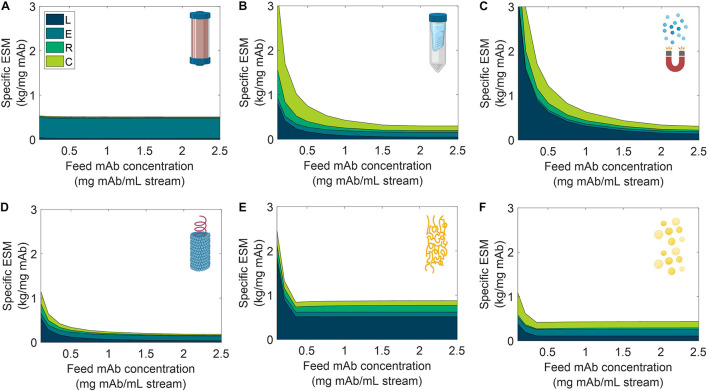
Specific equivalent system mass (per unit mass monoclonal antibody produced) broken down by labor (L), equipment (E), raw materials (R), and consumables (C) cost categories as a function of feed monoclonal antibody (mAb) concentration for **(A)** CHM, **(B)** SPN, **(C)** MAG, **(D)** VIN, **(E)** ELP, and **(F)** OLE. CHM, pre-packed chromatography; SPN, spin column; MAG, magnetic bead; VIN, plant virus-based nanoparticle; ELP, elastin-like polypeptide; OLE, oilbody-oleosin.

**FIGURE 7:**
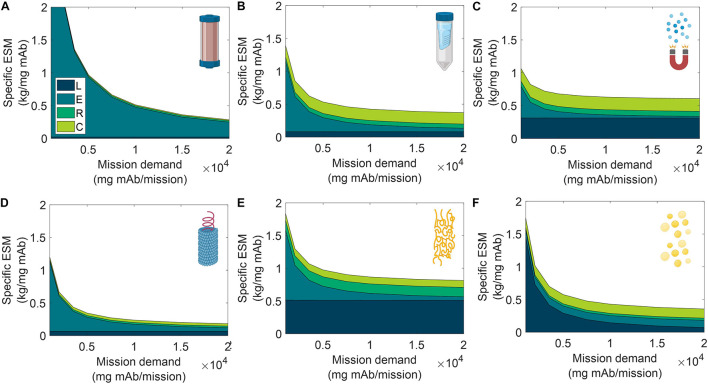
Specific equivalent system mass (per unit mass monoclonal antibody produced) broken down by labor (L), equipment (E), raw materials (R), and consumables (C) cost categories as a function of mission production demand for monoclonal antibody for **(A)** CHM, **(B)** SPN, **(C)** MAG, **(D)** VIN, **(E)** ELP, and **(F)** OLE. CHM, pre-packed chromatography; SPN, spin column; MAG, magnetic bead; VIN, plant virus-based nanoparticle; ELP, elastin-like polypeptide; OLE, oilbody-oleosin.

We analyzed the specific ESM output broken down by cost category for the six unit procedures over a range of input stream mAb concentrations (Figure 6) and mission demand for mAb (Figure 7). Specific ESM, termed cost of goods sold in traditional manufacturing analyses, is the ESM output required to produce 1 mg mAb. This is used in the scenario analyses to normalize ESM output across variation in mission demand for mAb. The optimal number of batches per mission was found and used for each unit procedure and scenario tested (Supplementary Tables 7, 8).

We observe the general and expected trends that specific ESM decreases with an increasing feed stream mAb concentration and mission demand. The CHM procedure exhibits notably limited sensitivity to feed stream mAb concentration, which can be attributed to the equipment-dominated cost profile, fixed column size, and nature of the governing reference protocol that does not specify restrictions on sample load volume. Depending on the pre-treatment of the feed stream, it may be more reasonable to impose constraints on the sample load volume. In contrast, the specific ESM output of the CHM procedure is the most sensitive to mission mAb demand with higher demand increasingly offsetting the fixed capital costs. The CHM procedure is also the largest capacity unit modeled in the analysis (i.e., CHM capacity is 30 mg mAb/unit as compared to 2.7 mg mAb/unit for MAG, the next highest capacity unit) and is accordingly expected to scale well with demand.

The SPN, ELP, OLE procedures exhibit behaviors in which the specific ESM output abruptly plateaus with an increasing feed stream mAb concentration. This observation can be attributed to the unit procedure operating in a mAb binding capacity-limited regime (as opposed to volume-limited for more dilute feeds) which also then controls and maintains unit procedure throughput (e.g., the ELP number of units, 37,044, and use cycles per mission, 2,058, is constant at and above 0.35 mg mAb/mL input stream concentration). This can be de-bottlenecked via technology (e.g., improved chemistry of the capture step unit leading to higher binding capacity) or methodology (e.g., increased concentration of the capture step unit leading to higher binding capacity) improvements.

Low demand scenarios are particularly relevant for examination in a space health context, as small capacity redundant and emergency utility is a likely proving ground for inclusion of a space pharmaceutical foundry. At the lower boundary of the tested range (1,000 mg mAb/mission), we see the ESM outputs from lowest to highest are re-ordered as MAG < VIN < SPN < OLE < ELP < CHM. Minimization of equipment costs are particularly important in this regime, and it is observed that, indeed, the ESM output near completely aligned with the ranking of equipment cost (MAG < VIN < SPN < ELP < OLE < CHM). It is likely that other non-ESM factors such as integration with other flown elements will understandably influence the design and composition of early and low capacity flown pharmaceutical foundries.

### Alternate Scenarios

#### Mission Configurations

**FIGURE 8:**
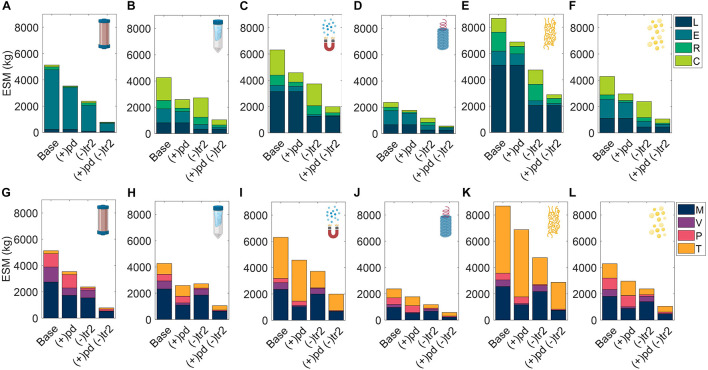
Evaluation of extended equivalent system mass values in various mission configurations broken down by labor (L), equipment (E), raw materials (R), and consumables (C) cost categories cost category and mass (M), volume (V), power (P), and labor time (T) constituents for CHM, **(A,G)**, SPN, **(B,H)**, MAG, **(C,I)**, VIN, **(D,J)**, ELP **(E,K)**, and OLE, **(F,L)**. Configurations include the base case scenario of manufacturing resources flown with the crew for pharmaceutical production on the surface and return transit (Base), and alternatives in which the manufacturing resources are flown prior to the crew in pre-deployment, (+)pd, the production window is limited to surface operations, (–)tr_2_, and a combination of the two previously stated alternatives, (+)pd (–)tr_2_. CHM, pre-packed chromatography; SPN, spin column; MAG, magnetic bead; VIN, plant virus-based nanoparticle; ELP, elastin-like polypeptide; OLE, oilbody-oleosin.

We explored variations to the base case RMA for all six unit procedures including scenarios in which the pharmaceutical manufacturing resources are shipped prior to the crew in pre-deployment, (+)pd, the production window has been truncated to close with the end of surface operations, (−)tr_2_, and a combination of the two prior modifications, (+)pd (−)tr_2_ (Figure 8). Costs of pre-deployment are included in the analyses and mission demand is kept constant regardless of the production window.

In all cases the ESM totals were reduced from the base case. Additionally, the general trend held that (−)tr_2_ scenario resulted in lower ESM totals than (+)pd scenario except for SPN, in which the increased raw material and consumable costs of (−)tr_2_ were sufficiently large to outweigh the reduction in equipment and labor costs of (+)pd. The combination (+)pd (−)tr_2_ scenario resulted in the lowest ESM totals at a fraction of the base case (as high as 39% reduction in SPN and as low as 21% reduction in ELP).

#### Equipment and Unit Throughput

Acknowledging the significance of the equipment capacity on ESM output, we further explored this contribution by comparing the base case ESM output of the centrifuge-utilizing procedures (SPN, VIN, ELP, and OLE) to that resulting from the use of alternative centrifuge models (Supplementary Table 9). This effectively results in a trade of equipment costs and batch throughput. The optimal number of batches per mission was found and used for each unit procedure and interval tested (Supplementary Table 10).

**FIGURE 9:**
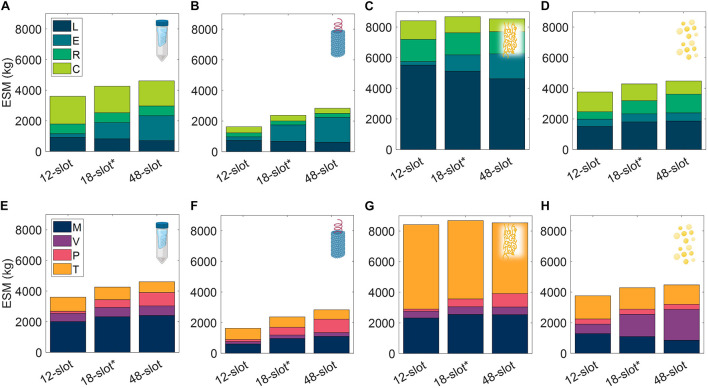
Changes in extended equivalent system mass values with different capacity centrifuge models broken down by labor (L), equipment (E), raw materials (R), and consumables (C) cost categories and mass (M), volume (V), power (P), and labor time (T) constituents for SPN **(A,E)**, VIN **(B,F)**, ELP **(C,G)**, and OLE **(D,H)**. SPN, spin column; VIN, plant virus-based nanoparticle; ELP, elastin-like polypeptide; OLE, oilbody-oleosin.

We observe in Figure 9 that the ESM values increased with the size of the centrifuge model, 12-slot < 18-slot (base) < 48-slot. The labor and consumables savings of higher batch throughput were outweighed by the higher equipment costs (including higher power costs). Operation duration is an important metric relevant to a pharmaceutical foundry that is not well reflected in ESM that is also impacted by this alternative scenario. The exception to this trend is the 48-slot condition for the ELP procedure, in which a lower consumable cost related to the number of use cycles per mission (i.e., pipette tips, tubes, and gloves) sufficiently lowered the total ESM below the 18-slot condition.

#### Technology Reusability

**FIGURE 10:**
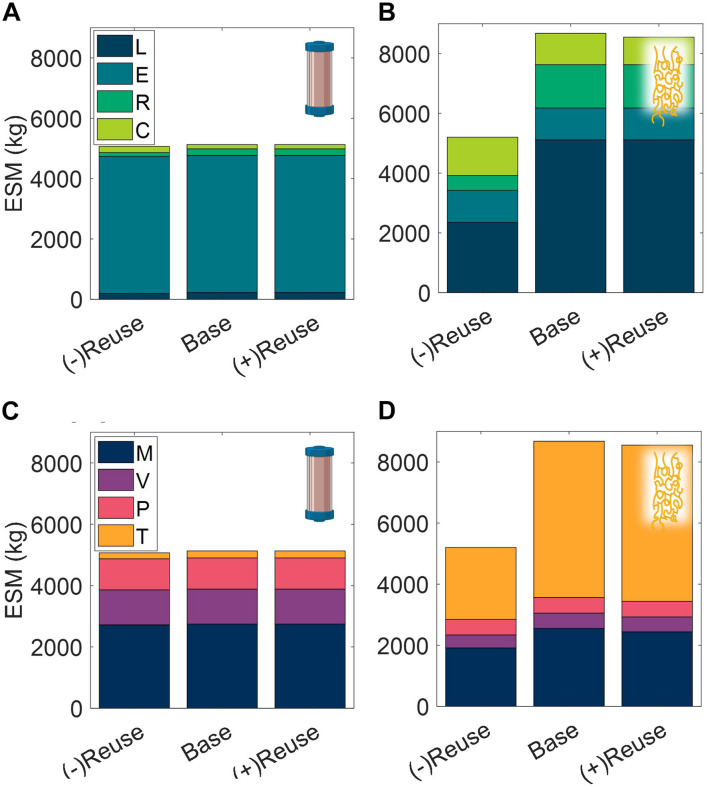
Changes in extended equivalent system mass values with reusability of purification technology broken down by labor (L), equipment (E), raw materials (R), and consumables (C) cost categories and mass (M), volume (V), power (P), and labor time (T) constituents for CHM **(A,C)**, and ELP **(B,D)**. (–)Reuse considers the technology as single-use and accordingly discards the unit procedure cleaning operations; (+) Reuse considers additional reuse cycles of the technology. CHM, pre-packed chromatography; ELP, elastin-like polypeptide.

The number of use cycles for liquid chromatography resins is an important economic parameter in commercial pharmaceutical manufacturing (Pathak and Rathore, 2016). Here we explore the impact of use cycles on the CHM and ELP procedures in a space mission context, looking at no reuse nor regeneration operation of the purification technology, (−)Reuse, and at an increased number of use cycles, (+)Reuse (Figure 10).

We observe that the terrestrial importance of use cycles does not prevail in this isolated ESM evaluation in a space context. The high purchase costs of resin are not considered in ESM and the impact of the reuse cycles is reduced to the mass and volume savings of the pre-packed column consumable. There is a minor decrease in ESM of the (+)Reuse over the base case scenario, but both of these result in substantially higher ESM than the (−)Reuse scenario, particularly for the ELP procedure, in which the regeneration operation has been removed in addition to the reusability of the technology.

These results echo the trend of single-use technology in commercial biotechnology in which manufacturers look to disposable plastic bioreactor and buffer bags as a means to reduce cleaning and validation costs (Shukla and Gottschalk, 2013). It would be valuable to further consider the utilization of single-use technology in a space pharmaceutical foundry, and in other space systems bioengineering applications, but it is important to point out the limited scope of this ESM analysis. Here we reiterate that the single unit procedure scope establishes a modular basis for pharmaceutical foundry ESM evaluation but does not realize the true circular economy advantages of reuse, which may be considerable for the regeneration step, and of biological systems for production of the purification reagent in general.

## Conclusion and Future Directions

In this study, we have introduced and applied the ESM framework to biopharmaceutical processing as a first step toward modeling and understanding the costs of Space Systems Bioengineering and, more specifically, of a long-duration space exploration medical foundry, which we believe may 1 day constitute a critical bioregenerative component of ECLSS for humans to be able to explore the surface of Mars. We have observed that the static behavior of ESM, while certainly maintaining usefulness in early stage analyses, may stymie later-stage analyses of bioregenerative life support technologies, which tend to behavior more dynamically than traditional abiotic counterparts. In the future, higher fidelity analyses may be performed using tools such as HabNet (Do et al., 2015), although the use of such dynamic mission design and modeling tools will require additional software engineering efforts. As it stands now, our techno-economic calculations both satisfy the three fundamental aspects for life support modeling (Jones, 2017) and provide helpful directions for future efforts to incorporate purification processes in space systems bioengineering.

The mAb affinity capture step represented an ideal starting point for biopharmaceutical purification cost analysis given the breadth of the mAb treatments for space-important health indications, the fact that mAb purification is considered a platform technology, and the diversity of affinity capture technologies. However, there are additional processing categories, such as size exclusion, ion exchange, and hydrophobic interaction unit procedures, which could be similarly studied in isolation for their general relevance in biopharmaceutical manufacturing. Establishing a unit procedure knowledge base for space-relevant economics of biopharmaceutical purification would provide additional benefit to the community.

We acknowledge that the ESM analysis performed in this study utilizes current Earth-based technologies, not Mars-designed processes, and that as technologies evolve and expand the analysis will need to be updated. The need to revisit and update ESM analyses periodically as technology develops is standard practice. This is well illustrated in a recent ESM analysis of plant lighting systems that compares solar fiber optics to photovoltaic-powered light emitting diode hybrid systems (Hardy et al., 2020). The study results reversed decade-old trade study outcomes in which solar fiber optics scored more favorably, citing rapid advances in solar photovoltaics and light emitting diode technologies.

Furthermore, the analysis presented does not encapsulate potentially significant characteristics of the unit procedures at the interfaces of the upstream and downstream biomanufacturing elements. For example, at the upstream interface the biotic unit procedures (VIN, ELP, and OLE) have been reported in literature to be effective capture mechanisms in “dirtier” feed solutions, perhaps absolving the need for more complex pre-capture clarification steps by virtue of process integration. At the downstream end, the eluate of the CHM unit procedure can be directly fed to the subsequent processing step, which would be particularly amenable for other column-based unit procedures, resulting in lower labor time and manufacturing duration. We also do not account for the uncertainty in performance associated with the developmental state of the technology. There have been substantially lower research and development investments in the biotic technologies than in the commercially available abiotic technologies; one may reasonably assume that there is more potential for improvements through biotic unit procedure optimization, while also considering that a larger driving force in abiotic unit procedure optimization for commercial terrestrial operations may balance or outweigh this. Forecasting on the technology development dynamics in the context of these, and other, forces could provide significant additional insights.

Several overarching lessons on the development required for deployment of pharmaceutical purification technology to support human health in space can be gleaned from the cost breakdown of the ESM framework employed in this study. The high mass costs for the mAb capture technologies investigated suggest strong incentives to pursue efforts in miniaturization to reduce not only equipment mass, but also reagent mass, as preparation for pharmaceutical foundries in space. The high labor costs and duration of some of the technologies studied likewise suggests that automatization of biopharmaceutical purification would be impactful. Automatization could also conceivably be valuable in reducing mass costs associated with manual manipulation, such as pipette tips and gloves, and those associated with ensuring sterile operation. We also underline the importance of scheduling and equipment sizing optimization; for example, the ESM penalty for capturing the mission demand of mAb with the VIN unit procedure yielded up to 40% higher total ESM for non-optimal scheduled manufacturing batches. Given the advantage of *in situ* manufacturing to respond to uncertainty in mission medicine demand, further research to explore scheduling and equipment sizing under uncertainty would provide valuable insight.

There are a series of challenges facing pharmaceutical foundries in space beyond processing. Perhaps the most daunting of these is the incompatibility of existing pharmaceutical regulatory compliance frameworks with the design constraints of *in situ* manufacturing. There are currently dozens to hundreds of analytical tests required to confirm process and product quality prior to release of the pharmaceutical for administration to human patients (Morrow and Felcone, 2004), which translates into a highly burdensome cost for *in situ* manufacturing of pharmaceuticals in space. Fortunately, there is a strong and parallel terrestrial need to reduce the burden of regulatory compliance while maintaining standards of quality assurance and control for personalized medicine, an individualized and patient-specific approach to medical care with widespread support. As mentioned earlier, trends of distributed and sustainable biomanufacturing on Earth provide additional support for reducing ESM-relevant costs.

The analyses presented in this study motivate future investigation into the ESM output of a complete pharmaceutical foundry for a more complete comparison to other ECLSS needs and subsequent formal evaluations of medical risk (i.e., loss of crew life, medical evacuation, crew health index, risk of radiation exposure-induced death from cancer) mitigation as a balance to the ESM costs. The Integrated Scalable Cyto-Technology system (Crowell et al., 2018), reported in literature as capable of “end-to-end production of hundreds to thousands of doses of clinical-quality protein biologics in about 3 d[ays],” is an automated and multiproduct pharmaceutical manufacturing system that may serve well as a starting point for a complete pharmaceutical foundry evaluation. While downstream costs are typically a large proportion of terrestrial biopharmaceutical production costs, they may represent an even higher proportion of the overall ESM costs. ESM is more closely aligned to PMI as a metric than to cost of goods sold in dollars, suggesting that downstream contributions to ESM may similarly dominate. Budzinski and team found that downstream operations contributed 82% of the total PMI for commercial mAb production (Budzinski et al., 2019).

Assembly of a complete pharmaceutical foundry ESM model would also enable investigation of more nuanced RMA design considerations, such as those relating to the influence of a fixed set, or anticipated probability distribution, of pharmaceutical product diversity and batch size on optimal system composition to meet given medical risk thresholds.

As stated in the original presentation of ESM theory and application, comparison of multiple approaches for a given subsystem with ESM, such as we are studying with the capture step of a mAb pharmaceutical foundry, should satisfy the same product quantity, product quality, reliability, and safety requirements (Levri et al., 2000). Of these assumptions, the product quality and safety requirements prove challenging for implementation in pharmaceutical foundry comparisons. It is worth noting that reliability is not considered in the scope of this preliminary study, given the varying technology readiness levels of the unit procedures, but that it should be included in future analyses of full purification schemes. By extension, the impact of microgravity and reduced gravity on reliability and unit operation performance, while not investigated in this study, is an important and complex consideration, that requires significant research to address. Similarly, stability of the production resources over the course of a mission duration should be further considered in future works. High product sensitivity to process changes, and the large battery of testing sometimes required to observe them (the extent of which will also change with the processes employed), creates a situation where ESM comparisons of pharmaceutical foundries that serve as technology decision making tools will absolutely need to meet this requirement, albeit at a considerable cost and/or complexity of execution.

The assessment of equivalent safety requirements, to the best of the knowledge of the authors, has been approached thus far in an *ad hoc* and qualitative manner, relying on extensive subject manner expertise and working process knowledge. One promising route to strengthening these critically important safety assessments would be to implement a formal assessment framework based on the environmental, health, and safety (EHS) assessment proposed by Biwer and Heinzle (2004), in which process inputs/outputs are ranked based on a series of hazard impact categories (e.g., acute toxicity, raw material availability, global warming potential) and impact groups (e.g., resources, organism). The key to a systematic space health-centric safety assessment like this is to establish space-relevant EHS impact categories (e.g., planetary protection, crew and ship safety). An improvement of the EHS underpinnings has the potential to provide significant benefits to future ESM analyses in the increasingly complex mission architecture of longer-duration missions.

## Data Availability Statement

The raw data supporting the conclusions of this article will be made available by the authors, without undue reservation.

## Author Contributions

MM, AB, SN, and KM designed the study. MM, AB, PN, LM, OH, and KY built the model used in the study. MM analyzed the data and wrote the first draft of the manuscript. All authors were involved in project discussion and interpretation and contributed to manuscript revision.

## Conflict of Interest

The authors declare that the research was conducted in the absence of any commercial or financial relationships that could be construed as a potential conflict of interest.

## Publisher’s Note

All claims expressed in this article are solely those of the authors and do not necessarily represent those of their affiliated organizations, or those of the publisher, the editors and the reviewers. Any product that may be evaluated in this article, or claim that may be made by its manufacturer, is not guaranteed or endorsed by the publisher.

## Footnotes

1
https://mars.nasa.gov/insight/mission/overview/

2
https://mars.nasa.gov/mars2020/mission/overview/

3
https://mars.nasa.gov/

4
http://go.nasa.gov/1VHDXxg

5
https://humanresearchroadmap.nasa.gov/Risks/

6
ondemandpharma.com

7
eqrx.com

8
kenup.eu

## Abbreviations

CHM: pre-packed chromatography
EHS: environmental, health, and safety
ELP: elastin-like polypeptide
ESM: equivalent system mass
Fc: fragment crystallizable
ISRU: *in situ* resource utilization
ISS: International Space Station
mAb: monoclonal antibody
MAG: magnetic bead
PMI: process mass intensity
OLE: oilbody-oleosin
RMA: reference mission architecture
SPN: spin column
VIN: plant virus-based nanoparticle.

## References

[B1] Adiga R. Al-adhami M. Andar A. Borhani S. Brown S. Burgenson D. (2018). Point-of-care production of therapeutic proteins of good-manufacturing-practice quality. *Nat. Biomed. Eng.* 2 675–686. 10.1038/s41551-018-0259-1 31015674

[B2] Afshinnekoo E. Scott R. T. MacKay M. J. Pariset E. Cekanaviciute E. Barker R. (2020). Fundamental biological features of spaceflight: advancing the field to enable deep-space exploration. *Cell* 183 1162–1184. 10.1016/j.cell.2020.10.050 33242416 PMC8441988

[B3] Anderson M. S. Ewert M. K. Keener J. F. (2018). *Life Support Baseline Values and Assumptions Document. NASA/TP-2015–218570/REV1.* Houston, TX: NASA.

[B4] Anselmo A. C. Gokarn Y. Mitragotri S. (2018). Non-invasive delivery strategies for biologics. *Nat. Rev. Drug Discov.* 18 19–40. 10.1038/nrd.2018.183 30498202

[B5] Antonsen E. Bayuse T. Blue R. Daniels V. Hailey M. Hussey S. (2017). *Evidence Report: Risk of Adverse Health Outcomes and Decrements in Performance due to In-Flight Medical Conditions Human Research Program Exploration Medical Capabilities Element Approved for Public Release.* Houston, TX: NASA.

[B6] Bell M. R. Engleka M. J. Malik A. Strickler J. E. (2013). To fuse or not to fuse: what is your purpose? *Protein Sci.* 22 1466–1477. 10.1002/pro.2356 24038604 PMC3831663

[B7] Berliner A. J. Hilzinger J. M. Abel A. J. Mcnulty M. Makrygiorgos G. Averesch N. J. H. (2020). Towards a biomanufactory on mars. *Preprint* 10.20944/preprints202012.0714.v1 32283112

[B8] Berliner A. J. Makrygiorgos G. Hill A. (2021). Extension of Equivalent System Mass for Human Exploration missions on mars. *Preprint* 10.20944/preprints202101.0363.v1 35918365 PMC9345954

[B9] Biwer A. Heinzle E. (2004). Environmental assessment in early process development. *J. Chem. Technol. Biotechnol.* 79 597–609. 10.1002/jctb.1027

[B10] Bizzarri M. Masiello M. G. Cucina A. Guzzi R. (2017). Journey to mars: a biomedical challenge. Perspective on future human space flight. *J. Biol. Sci. hypotheses Opin.* 1 15–26. 10.13133/2532-5876_2.6

[B11] Blue R. S. Bayuse T. M. Daniels V. R. Wotring V. E. Suresh R. Mulcahy R. A. (2019). Supplying a pharmacy for NASA exploration spaceflight: challenges and current understanding. *NPJ Microgravity* 5:14. 10.1038/s41526-019-0075-2 31231676 PMC6565689

[B12] Budzinski K. Blewis M. Dahlin P. D’Aquila D. Esparza J. Gavin J. (2019). Introduction of a process mass intensity metric for biologics. *Nat. Biotechnol.* 49 37–42. 10.1016/j.nbt.2018.07.005 30121383

[B13] Choe W. Durgannavar T. Chung S. (2016). Fc-binding ligands of immunoglobulin G: an overview of high affinity proteins and peptides. *Materials (Basel)* 9:994. 10.3390/ma9120994 28774114 PMC5456964

[B14] Crowell L. E. Lu A. E. Love K. R. Stockdale A. Timmick S. M. Wu D. (2018). On-demand manufacturing of clinical-quality biopharmaceuticals. *Nat. Biotechnol.* 36:988. 10.1038/nbt.4262 30272677 PMC6443493

[B15] Cytiva (2006). *Protein A HP SpinTrap Product Booklet.* Available online at: https://www.cytivalifesciences.co.jp/tech_support/manual/pdf/28906770.pdf (accessed March 28, 2021).

[B16] Cytiva (2020). *Protein A Mag Sepharose Xtra Protein G Mag Sepharose Xtra Affinity Chromatography instructions for Use.* Available online at: https://cdn.cytivalifesciences.com/dmm3bwsv3/AssetStream.aspx?mediaformatid=10061&destinationid=10016&assetid=15782 (accessed March 28, 2021).

[B17] Cytiva (2021). *Affinity Chromatography Handbook, Vol. 1: Antibodies.* Available online at: https://cdn.cytivalifesciences.com/dmm3bwsv3/AssetStream.aspx?mediaformatid=10061&destinationid=10016&assetid=11660 (accessed March 28, 2021).

[B18] Do S. Owens A. de Weck O. (2015). “HabNet – an integrated habitation and supportability architecting and analysis environment,” in *Proceedings of the 45th International Conference on Environmental Systems*, (Bellevue).

[B19] Drake B. G. Hoffman S. J. Beaty D. W. (2010). “Human exploration of mars, design reference architecture 5.0,” in *Proceedings of the IEEE Aerospace Conference Proceedings*, (Big Sky, MT), 10.1109/AERO.2010.5446736

[B20] Drake B. G. Watts K. D. (2014). *Human Exploration of Mars Design Reference Architecture 5.0 Addendum #2.* Available online at: http://www.sti.nasa.gov (accessed March 7, 2021).

[B21] Du B. Daniels V. R. Vaksman Z. Boyd J. L. Crady C. Putcha L. (2011). Evaluation of physical and chemical changes in pharmaceuticals flown on space missions. *AAPS J.* 13 299–308. 10.1208/s12248-011-9270-0 21479701 PMC3085701

[B22] Faienza M. F. Chiarito M. D’amato G. Colaianni G. Colucci S. Grano M. (2018). Monoclonal antibodies for treating osteoporosis. *Expert Opin. Biol. Ther.* 18 149–157. 10.1080/14712598.2018.1401607 29113523

[B23] Goding J. W. (1996). *Monoclonal Antibodies*, 3rd Edn. Cambridge, MA: Academic Press.

[B24] Haile L. A. Puig M. Kelley-Baker L. Verthelyi D. (2015). Detection of innate immune response modulating impurities in therapeutic proteins. *PLoS One* 10:e0125078. 10.1371/journal.pone.0125078 25901912 PMC4406594

[B25] Hardy J. M. Kusuma P. Bugbee B. Wheeler R. Ewert M. (2020). “Providing photons for food in regenerative life support: a comparative analysis of solar fiber optic and electric light systems,” in *Proceedings of the 2020 International Conference on Environmental Systems*, (Logan, UT).

[B26] Harrison R. G. Todd P. W. Rudge S. R. Petrides D. P. (2015). in *Bioseparations Science and Engineering*, 2nd Edn, eds Gubbins K. E. Barteau M. A. Lauffenburger D. A. Morari M. Ray W. H. Russel W. B. (Oxford: Oxford University Press).

[B27] Hendrickx L. De Wever H. Hermans V. Mastroleo F. Morin N. Wilmotte A. (2006). Microbial ecology of the closed artificial ecosystem MELiSSA (Micro-Ecological Life Support System Alternative): reinventing and compartmentalizing the Earth’s food and oxygen regeneration system for long-haul space exploration missions. *Res. Microbiol.* 157 77–86. 10.1016/j.resmic.2005.06.014 16431089

[B28] Hogan J. A. Kang S. Cavazzoni J. Levri J. A. Finn C. Luna B. (2000). *A Simulation Study Comparing Incineration and Composting in a Mars-Based Advanced Life Support System NASA Document ID 20000121172.* Moffett Field, CA: NASA.

[B29] Hua L. Hilliard J. J. Shi Y. Tkaczyk C. Cheng L. I. Yu X. (2014). Assessment of an anti-alpha-toxin monoclonal antibody for prevention and treatment of *Staphylococcus aureus*-induced pneumonia. *Antimicrob. Agents Chemother.* 58 1108–1117. 10.1128/AAC.02190-13 24295977 PMC3910899

[B30] Iino Y. Takahashi E. Ida S. Kikuchi S. (2019). Clinical efficacy of anti-IL-5 monoclonal antibody mepolizumab in the treatment of eosinophilic otitis media. *Auris Nasus Larynx* 46 196–203. 10.1016/j.anl.2018.07.011 30122651

[B31] Jones H. (2017). “How should life support be modeled and simulated?,” in *Proccedings of the 47th International Conference on Environmental Systems*, (Charleston. BC).

[B32] Jones H. W. (2001). *The cost and Equivalent System Mass of Space Crew Time SAE Technical Paper 2001-01-2359.* Warrendale PA: SAE International, 10.4271/2001-01-2359

[B33] Jugler C. Joensuu J. Chen Q. (2020). Hydrophobin-protein a fusion protein produced in plants efficiently purified an anti-west nile virus monoclonal antibody from plant extracts via aqueous two-phase separation. *Int. J. Mol. Sci.* 21:2140. 10.3390/ijms21062140 32244994 PMC7139538

[B34] Kang T. H. Jung S. T. (2019). Boosting therapeutic potency of antibodies by taming Fc domain functions. *Exp. Mol. Med.* 51:138. 10.1038/s12276-019-0345-9 31735912 PMC6859160

[B35] Krawczyk A. Dirks M. Kasper M. Buch A. Dittmer U. Giebel B. (2015). Prevention of herpes simplex virus induced stromal keratitis by a glycoprotein b-specific monoclonal antibody. *PLoS One* 10:e0116800. 10.1371/journal.pone.0116800 25587898 PMC4294644

[B36] Kwon K.-C. Daniell H. (2015). Low-cost oral delivery of protein drugs bioencapsulated in plant cells. *Plant Biotechnol. J.* 13 1017–1022. 10.1111/pbi.12462 26333301 PMC4769795

[B37] Laptoš T. Omersel J. (2018). The importance of handling high-value biologicals: physico-chemical instability and immunogenicity of monoclonal antibodies. *Exp. Ther. Med.* 15 3161. 10.3892/ETM.2018.5821 29556253 PMC5841069

[B38] Levri J. A. Fisher J. W. Jones H. W. Drysdale A. E. Ewert M. K. Hanford A. J. (2003). *Advanced Life Support Equivalent System Mass Guidelines Document ALS Equivalent System Mass Guidelines DocumentNASA/TM-2003-212278.* Moffett Field, CA: NASA.

[B39] Levri J. A. Vaccari D. A. Drysdale A. E. (2000). *Theory and Application of the Equivalent System Mass Metric Technical Paper 2000-01-2395.* Warrendale, PA: SAE International, 10.4271/2000-01-2395

[B40] McLean M. D. Chen R. Yu D. Mah K.-Z. Teat J. Wang H. (2012). Purification of the therapeutic antibody trastuzumab from genetically modified plants using safflower Protein A-oleosin oilbody technology. *Transgenic Res.* 21 1291–1301. 10.1007/s11248-012-9603-5 22382463

[B41] McNulty M. J. Xiong Y. M. Yates K. Karuppanan K. Hilzinger J. M. Berliner A. J. (2021). Molecular pharming to support human life on the moon, mars, and beyond. *Crit. Rev. Biotechnol.* 41 849–864. 10.1080/07388551.2021.1888070 33715563

[B42] Menezes A. A. Cumbers J. Hogan J. A. Arkin A. P. (2015). Towards synthetic biological approaches to resource utilization on space missions. *J. R. Soc. Interface* 12:20140715. 10.1098/rsif.2014.0715 25376875 PMC4277073

[B43] Millet L. J. Lucheon J. D. Standaert R. F. Retterer S. T. Doktycz M. J. (2015). Modular microfluidics for point-of-care protein purifications. *Lab Chip* 15 1799–1811. 10.1039/c5lc00094g 25740172

[B44] Mitragotri S. Burke P. A. Langer R. (2014). Overcoming the challenges in administering biopharmaceuticals: formulation and delivery strategies. *Nat. Rev. Drug Discov.* 13 655–672. 10.1038/nrd4363 25103255 PMC4455970

[B45] Morrow T. Felcone L. H. (2004). Defining the difference: what Makes Biologics Unique. *Biotechnol. Healthc.* 1 24–29. PMC3564302 23393437

[B46] Murphy T. W. Sheng J. Naler L. B. Feng X. Lu C. (2019). On-chip manufacturing of synthetic proteins for point-of-care therapeutics. *Microsystems Nanoeng.* 5 1–12. 10.1038/s41378-019-0051-8 31057940 PMC6431678

[B47] Musk E. (2017). Making humans a multi-planetary species. *New Sp.* 5 46–61. 10.1089/space.2017.29009.emu

[B48] Pathak M. Rathore A. S. (2016). Mechanistic understanding of fouling of protein A chromatography resin. *J. Chromatogr. A* 1459 78–88. 10.1016/j.chroma.2016.06.084 27423774

[B49] Pollard D. Brower M. Richardson D. (2017). “Progress toward automated single-use continuous monoclonal antibody manufacturing via the protein refinery operations lab,” in *Continuous Biomanufacturing - Innovative Technologies and Methods*, ed Subramanian G. (Weinheim: Wiley-VCH Verlag GmbH & Co. KGaA), 107–130. 10.1002/9783527699902.ch4

[B50] Ramos-de-la-Peña A. M. González-Valdez J. Aguilar O. (2019). Protein A chromatography: challenges and progress in the purification of monoclonal antibodies. *J. Sep. Sci.* 42 1816–1827. 10.1002/jssc.201800963 30811843

[B51] Rathore A. S. Pathak M. Ma G. Bracewell D. G. (2015). Re-use of Protein A resin: fouling and economics. *BioPharm Int.* 3:2015.

[B52] Rodríguez-Ruiz I. Babenko V. Martínez-Rodríguez S. Gavira J. A. (2018). Protein separation under a microfluidic regime. *Analyst* 143 606–619. 10.1039/c7an01568b 29214270

[B53] Schuster N. M. Rapoport A. M. (2016). New strategies for the treatment and prevention of primary headache disorders. *Nat. Rev. Neurol.* 12 635–650. 10.1038/nrneurol.2016.143 27786243

[B54] Sheldon R. A. (2007). The E Factor: fifteen years on. *Green Chem.* 9 1273–1283. 10.1039/b713736m

[B55] Sheth R. D. Jin M. Bhut B. V. Li Z. Chen W. Cramer S. M. (2014). Affinity precipitation of a monoclonal antibody from an industrial harvest feedstock using an ELP-Z stimuli responsive biopolymer. *Biotechnol. Bioeng.* 111 1595–1603. 10.1002/bit.25230 24595842

[B56] Shukla A. A. Gottschalk U. (2013). Single-use disposable technologies for biopharmaceutical manufacturing. *Trends Biotechnol.* 31 147–154. 10.1016/j.tibtech.2012.10.004 23178074

[B57] Škalko-Basnet N. (2014). Biologics: the role of delivery systems in improved therapy. *Biol. Targets Ther.* 8 107–114. 10.2147/BTT.S38387 24672225 PMC3964020

[B58] Sommerfeld S. Strube J. (2005). Challenges in biotechnology production - Generic processes and process optimization for monoclonal antibodies. *Chem. Eng. Process Process Intensif.* 44 1123–1137. 10.1016/j.cep.2005.03.006

[B59] Steinebach F. Karst D. Morbidelli M. (2017). “Design of integrated continuous processes for high-quality biotherapeutics,” in *Continuous Biomanufacturing - Innovative Technologies and Methods*, ed Subramanian G. (Weinheim: Wiley-VCH Verlag GmbH & Co. KGaA), 457–480. 10.1002/9783527699902.ch16

[B60] Steven Siceloff K. (2008). *NASA - Recycling Water is not Just for Earth Anymore.* Available online at: https://www.nasa.gov/mission_pages/station/behindscenes/waterrecycler.html (Accessed April 19, 2021).

[B61] Strube J. Ditz R. Kornecki M. Huter M. Schmidt A. Thiess H. (2018). Process intensification in biologics manufacturing. *Chem. Eng. Process. Process Intensif.* 133 278–293. 10.1016/J.CEP.2018.09.022

[B62] Sykes C. (2018). Time- and temperature-controlled transport: supply chain challenges and solutions. *P T* 43 154–170. 29491697 PMC5821242

[B63] Tylecote A. (2019). Biotechnology as a new techno-economic paradigm that will help drive the world economy and mitigate climate change. *Res. Policy* 48 858–868. 10.1016/j.respol.2018.10.001

[B64] Uhde-Holzem K. McBurney M. Tiu B. D. B. Advincula R. C. Fischer R. Commandeur U. (2016). Production of immunoabsorbent nanoparticles by displaying single-domain protein a on potato virus X. *Macromol. Biosci.* 16 231–241. 10.1002/mabi.201500280 26440117

[B65] Ulrich S. Ebel F. (2020). Monoclonal antibodies as tools to combat fungal infections. *J. Fungi* 6 22. 10.3390/jof6010022 32033168 PMC7151206

[B66] Vickery B. P. Vereda A. Casale T. B. Beyer K. du Toit G. O’B Hourihane J. (2018). AR101 oral immunotherapy for peanut allergy. *N. Engl. J. Med.* 379 1991–2001. 10.1056/NEJMoa1812856 30449234

[B67] Wall M. (2011). *NASA’s Shuttle Program Cost $209 Billion - Was it Worth It? | Space. Space.com.* Available online at: https://www.space.com/12166-space-shuttle-program-cost-promises-209-billion.html (accessed October 3, 2021)

[B68] Werner S. Marillonnet S. Hause G. Klimyuk V. Gleba Y. (2006). Immunoabsorbent nanoparticles based on a tobamovirus displaying protein A. *Proc. Natl. Acad. Sci. U.S.A.* 103 17678–17683. 10.1073/pnas.0608869103 17090664 PMC1635023

[B69] Whealan George K. (2019). The economic impacts of the commercial space industry. *Space Policy* 47 181–186. 10.1016/j.spacepol.2018.12.003

[B70] Wotring V. (2018). “Space pharmacology: how space affects pharmacology,” in *Drug Discovery and Evaluation: Methods in Clinical Pharmacology*, eds Hock F. Gralinski M. (Cham: Springer International Publishing), 1–13. 10.1007/978-3-319-56637-5_68-1

[B71] Zabel P. (2020). Influence of crop cultivation conditions on space greenhouse equivalent system mass. *CEAS Sp. J.* 13 3–15. 10.1007/s12567-020-00317-5

[B72] Zahavi D. Weiner L. (2020). Monoclonal antibodies in cancer therapy. *Antibodies* 9:34. 10.3390/antib9030034 32698317 PMC7551545

[B73] Zhao J. Wang Y. Xu C. Liu K. Wang Y. Chen L. (2017). Therapeutic potential of an anti-high mobility group box-1 monoclonal antibody in epilepsy. *Brain Behav. Immun.* 64 308–319. 10.1016/j.bbi.2017.02.002 28167116

