# Supplementary material for: Evaluating the Cost of Pharmaceutical Purification for a Long-Duration Space Exploration Medical Foundry
Source: Front Microbiol. 2021 Oct 11;12:700863. doi: 10.3389/fmicb.2021.700863 (PMC8542935; doi:10.3389/fmicb.2021.700863)
Supplement: Supplementary file 1 [file Data_Sheet_1.docx]

Supplementary Material

**Supplementary Table S1.** Example commercially approved monoclonal antibody (mAb) therapies of relevance to human health in space that have been considered in the determination of the reference mission pharmaceutical demand. Need basis is defined per the listed indication and FDA label. Demand estimates are derived by multiplying the FDA-approved need basis by the crew size and the duration of the demand. Asterisk (*) denotes an antibody drug conjugate.

| mAb | Indication | Dose | Need Basis |
| --- | --- | --- | --- |
| Erenumab-aooe ([FDA Label](https://www.accessdata.fda.gov/drugsatfda_docs/label/2018/761077s000lbl.pdf)) | Migraine headache prevention | 70 mg (or 140 mg) | 1 dose/month |
| Romosozumab ([FDA Label](https://www.accessdata.fda.gov/drugsatfda_docs/label/2019/761062s000lbl.pdf)) | Bone regeneration | 210 mg | 1 dose/month |
| Gemtuzumab ozogamicin* ([FDA Label](https://www.accessdata.fda.gov/drugsatfda_docs/label/2017/761060lbl.pdf)) | Acute myeloid leukemia | 6 mg/m^2^; 3 mg/m^2^; 2 mg/m^2^ | day 1/day 8/every 4 weeks; 1 course/year |

**
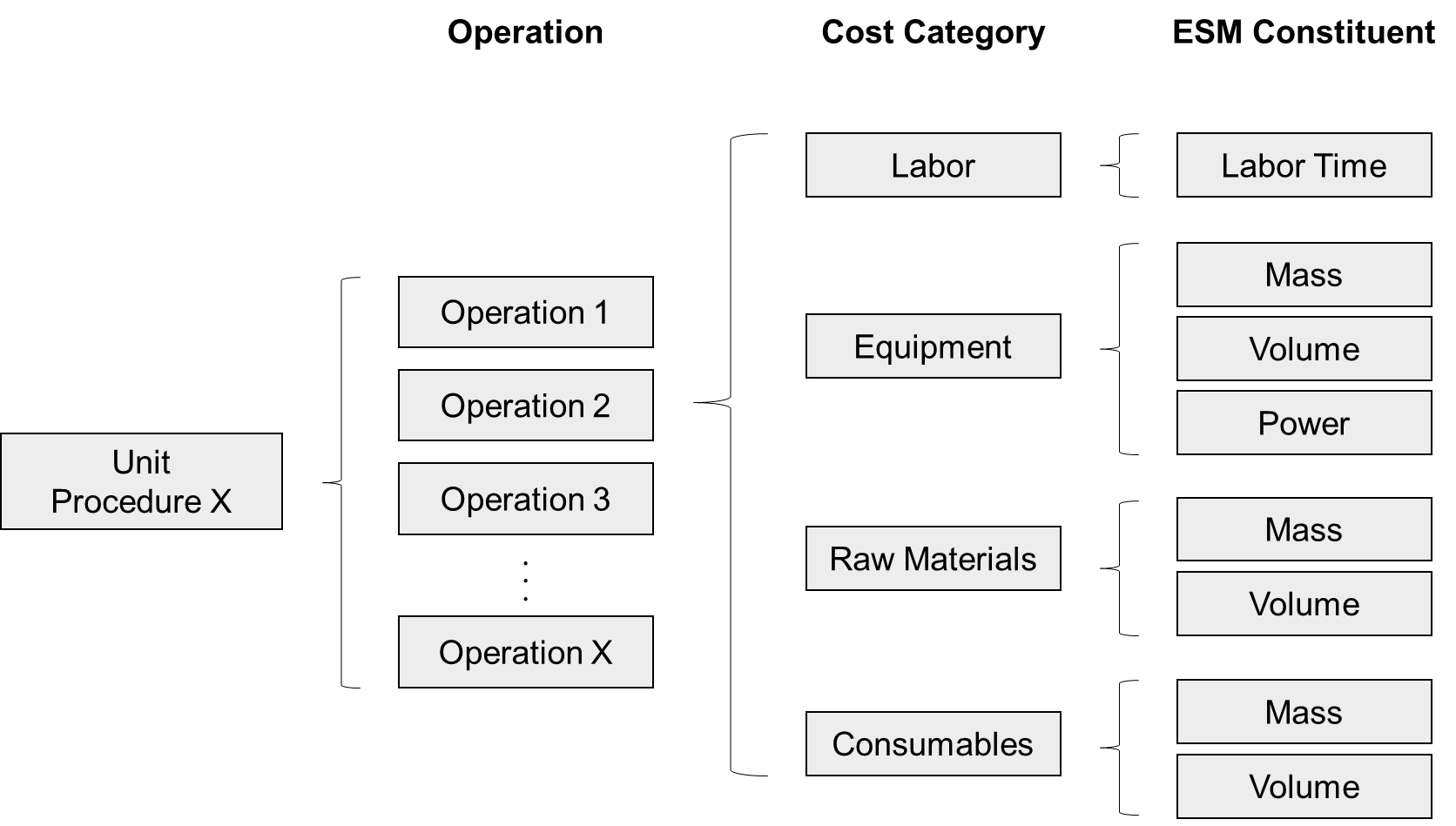
**

**Supplementary Figure S1.** Schematic of deterministic unit procedure model construction grouped by operation, cost category, and equivalent system mass (ESM) constituent.

**Supplementary Table S2.** Unit procedure assumptions for maximal feed stream volume and monoclonal antibody (mAb) binding capacity.

| Unit Procedure | Code | mAb Binding Capacity | Maximal Feed Stream Volume |
| --- | --- | --- | --- |
| Pre-packed chromatography | CHM | 30 mg/mL resin | N/A |
| Spin column | SPN | 1 mg/column | 0.6 mL |
| Magnetic bead | MAG | 27 mg/mL bead slurry | 0.3 mL |
| Plant virus-based nanoparticle | VIN | 4 mg/mL stock solution | 2 mL* |
| Elastin-like polypeptide | ELP | 0.42 mg/mL stock solution | 0.8 mL*^,¥^ |
| Oilbody-oleosin | OLE | 6.74 mg/mL stock solution | 0.1 mL*^,γ^ |

* based on 2 mL unit volume; actual feed stream volume added is based on the amount of stock solution required and thus mAb quantity in the feed stream.
^¥^ reduced from 2 mL maximal to account for volume needed for salt solution addition (0.4 mL) and required 1:1 volume ratio of ELP:mAb.
γ reduced from 2 mL maximal to account for required 1:20 volume ratio of OLE:mAb.

**Supplementary Table S3.** Labor time standardizations applied to common operations across unit procedures.

| Operation | Value | Unit |
| --- | --- | --- |
| Monitoring | 0.05 | labor hour/hour |
| Preparation  (incubation + centrifugation) | 1.0 | min/effective batch |
| Pipetting liquid | 0.5 | min/solution type |
|  | 0.1 | min/additional unit/effective batch |
| Resuspending pellet | 1 | min/unit |

**Supplementary Table S4.** Equivalency factor values used to generate equivalent system mass values from constituents of mass, volume, power, cooling, and labor.

| Segment | L_eq_ (kg/kg) | M_eq_ (kg/kg) | V_eq_ (kg/m^3^) | P_eq_ (kg/kW) | C_eq_  (kg/kW) | T_eq_  (kg/CM-h) |
| --- | --- | --- | --- | --- | --- | --- |
| Pre-deployment (Pd) | 2.77 | 1 | 9.16 | 237 | 40 | 0.7 |
| Transit to Mars (Tr1) | 10 | 1 | 133.8 | 136 | 50 | 0.7 |
| Surface Operation (Su) | 1 | 1 | 9.16 | 228 | 145 | 0.7 |
| Return Transit (Tr2) | 10 | 1 | 133.8 | 136 | 50 | 0.7 |

**Supplementary Table S5.** Optimal number of batches per mission in the base case scenario for each unit procedure, as determined via minimization of equivalent system mass.

| Unit procedure | Optimal number of batches per mission |
| --- | --- |
| CHM | 342 |
| SPN | 948 |
| MAG | 5670 |
| VIN | 360 |
| ELP | 2058 |
| OLE | 846 |

**Supplementary Table S6.** Mars surface mission equivalency factor values used by Zabel, 2020 of a space greenhouse.

| Segment | M_eq_ (kg/kg) | V_eq_ (kg/m^3^) | P_eq_ (kg/kW) | C_eq_  (kg/kW) | T_eq_  (kg/CM-h) |
| --- | --- | --- | --- | --- | --- |
| Surface Operation (Su) | 1.0 | 215.5 | 87.0 | 146.0 | 0.465 |

**Supplementary Table S7.** Optimal number of effective batches per mission in the mAb stream composition scenario analysis conditions for each unit procedure, as determined via minimization of equivalent system mass.

| mg/mL | 0.10 | 0.20 | 0.35 | 0.50 | 075 | 1.00 | 1.50 | 2.00 | 5.00 |
| --- | --- | --- | --- | --- | --- | --- | --- | --- | --- |
| CHM | 342 | 342 | 342 | 342 | 342 | 342 | 342 | 342 | 342 |
| SPN | 9450 | 4728 | 2700 | 1896 | 1260 | 948 | 630 | 568 | 568 |
| MAG | 56694 | 28350 | 16200 | 11340 | 7560 | 5670 | 3780 | 2838 | 1134 |
| VIN | 2910 | 1494 | 882 | 639 | 450 | 360 | 264 | 216 | 132 |
| ELP | 7092 | 3546 | 2058 | 2058 | 2058 | 2058 | 2058 | 2058 | 2058 |
| OLE | 2988 | 1494 | 854 | 846 | 846 | 846 | 846 | 846 | 846 |

**Supplementary Table S8.** Optimal number of batches per mission in the mAb demand scenario analysis conditions for each unit procedure, as determined via minimization of equivalent system mass.

| Demand x 10^3^ (mg mAb/mission) | 1.0 | 2.0 | 3.5 | 5.0 | 7.5 | 10.0 | 15.0 | 20.0 | 30.0 |
| --- | --- | --- | --- | --- | --- | --- | --- | --- | --- |
| CHM | 36 | 70 | 120 | 172 | 257 | 342 | 512 | 682 | 1022 |
| SPN | 96 | 192 | 336 | 474 | 714 | 948 | 1422 | 1890 | 2844 |
| MAG | 568 | 1134 | 1988 | 2838 | 4260 | 5670 | 8508 | 11340 | 17010 |
| VIN | 36 | 72 | 126 | 180 | 270 | 360 | 533 | 714 | 1068 |
| ELP | 210 | 414 | 726 | 1032 | 1548 | 2058 | 3090 | 4116 | 6174 |
| OLE | 86 | 170 | 300 | 422 | 632 | 846 | 1264 | 1692 | 2532 |

**Supplementary Table S9.** List of centrifuge models used in the alternative centrifuge scenario.

| Model | Vendor | Capacity | Mass (kg) | Dimensions (cm) | Power (kW) |
| --- | --- | --- | --- | --- | --- |
| MiniSpin | Eppendorf | 12 | 3.7 | 22.5 x 23.0 x 13.0 | 0.085 |
| 5418R | Eppendorf | 18 | 22 | 0.0345 | 0.320 |
| 5427R | Eppendorf | 48 | 30 | 31.9 x 54.0 x 25.4 | 0.550 |

**Supplementary Table S10.** Optimal number of effective batches per mission in the centrifuge model alternative scenario conditions for each analyzed unit procedure, as determined via minimization of equivalent system mass.

| Centrifuge model | MiniSpin | 5418R | 5427R |
| --- | --- | --- | --- |
| SPN | 1422 | 948 | 360 |
| VIN | 533 | 360 | 137 |
| ELP | 3090 | 2058 | 774 |
| OLE | 1264 | 846 | 317 |
